# Cooperative enhancement of deoxyribozyme activity by chemical modification and added cationic copolymer

**DOI:** 10.1080/14686996.2016.1208627

**Published:** 2016-07-29

**Authors:** Ken Saito, Naohiko Shimada, Atushi Maruyama

**Affiliations:** ^a^ Department of Life Science and Technology, Tokyo Institute of Technology, Yokohama, Japan

**Keywords:** Deoxyribozyme, Cationic copolymer, Locked Nucleic Acid, 2’ O-Methyl RNA, Turnover reaction, 30 Bio-inspired and biomedical materials, 101 Self-assembly / Self-organized materials, 211 Scaffold/Tissue engineering/Drug delivery

## Abstract

Deoxyribozymes (DNAzymes) having RNA-cleaving activity have widely been explored as tools for therapeutic and diagnostic purposes. Both the chemical cleaving step and the turnover step should be improved for enhancing overall activity of DNAzymes. We have shown that cationic copolymer enhanced DNAzyme activity by increasing turnover efficacy. In this paper, effectｓ of the copolymer on DNAzymes modified with locked nucleic acids (LNA) or 2′-*O*-methylated (2′-OMe) nucleic acids were studied. The copolymer increased activity of these chemically modified DNAzymes. More than 30-fold enhancement in multiple-turnover catalytic activity was observed with 2′-OMe-modified DNAzyme in the presence of the copolymer. DNAzyme catalytic activity was successfully enhanced by cooperation of the added copolymer and chemical modification of DNAzyme.

## Graphical abstract

**Figure:**
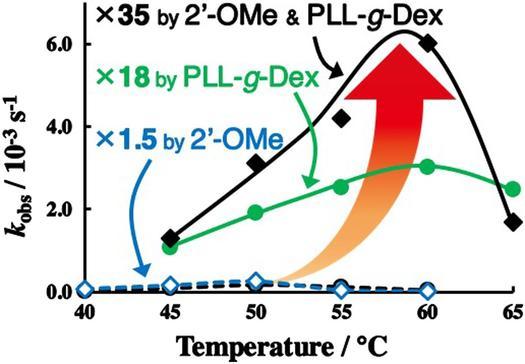

## 1. Introduction

Deoxyribozymes (DNAzymes) are DNA sequences having catalytic activities.[1–3] DNAzymes have several advantages over ribozymes or protein enzymes. DNAzymes are chemically and biologically more stable than ribozymes and protein enzymes, allowing ease in handling and storage. DNAzymes are readily prepared and modified. The 10–23 DNAzyme has catalytic activity to cleave RNA with sequence-specific manner.[4–7] Its activity exceeds that of any other known nucleic acid enzymes. 10–23 DNAzyme has been extensively studied for biomedical applications, such as nucleic acid medicines and genetic analyses.[8–12] However, the catalytic activity should be improved to satisfy these applications.

We have reported that cationic comb-type copolymers, PLL-*g*-Dex, consisting of a polycationic backbone and water soluble grafts facilitated hybridization of nucleic acids by reducing electrostatic repulsion between nucleic acid strands.[13–17] We recently reported that the copolymer accelerated multiple-turnover reactivity of 10–23 DNAzyme [18] and its derivative, multicomponent nucleotide enzyme (MNAzyme).[19]

A variety of chemically modified nucleic acids was explored to improve hybridization properties of nucleic acids. Locked nucleic acid (LNA),[20,21] and 2′-*O*-methylated (2′-OMe) nucleic acids [22,23] convey an RNA-like character to the DNA strands inserted with these modified nucleic acids. Enhanced activity of DNAzyme by insertion of chemically modified nucleic acids to substrate-binding arms of DNAzyme was reported.[24–26]

To improve further the DNAzyme reactivity, we are interested in examining the cooperation of the added cationic comb-type copolymer in the reaction of the chemically modified DNAzymes. In this study, catalytic activities of 10–23 DNAzyme modified with LNA or 2′-OMe were evaluated in the absence and presence of PLL-*g*-Dex. While 2′-OMe modification and the copolymer showed 1.5-fold and 17-fold enhancements, respectively, 30-fold enhancement of DNAzyme activity by this modification was observed in the presence of the copolymer. The result clearly demonstrated that successful cooperation of the added copolymer and chemical modification of DNAzyme.

## 2. Materials and methods

### 2.1. Materials

**Figure 1.**
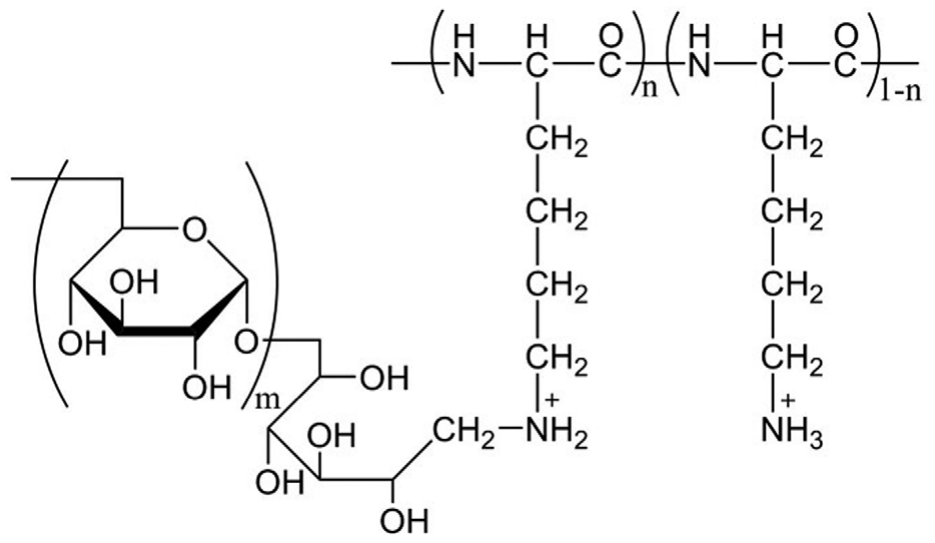
Structural formula of the cationic comb-type copolymer PLL-*g*-Dex. *M*_w_ of the PLL backbone: 7.5 × 10^3^, *M*_w_ of dextran grafts: 1.0 × 10^4^, grafting degree of dextran: 11.5 mol.%.

**Figure 2.**
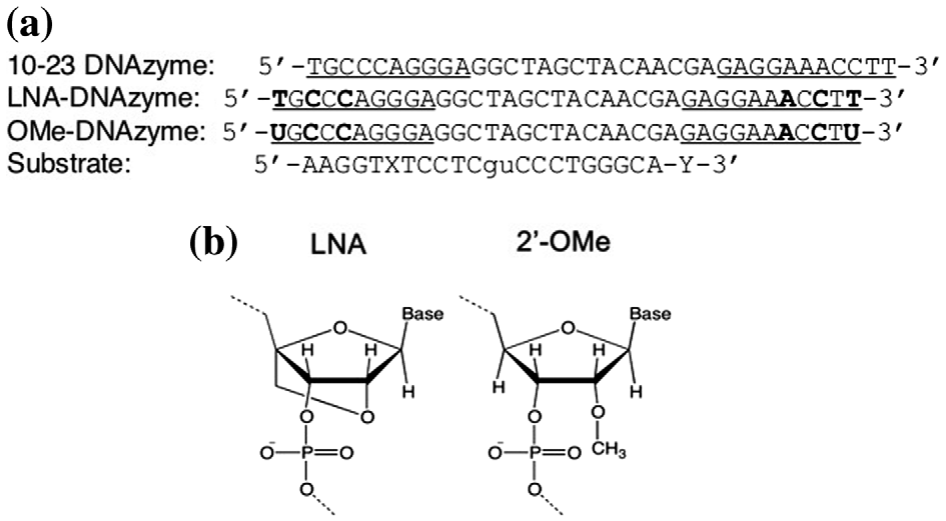
(a) Oligonucleotide sequences used in this study. LNA or 2′-OMe modified nucleotides are represented with bold fonts. RNA are represented with small letters. X and Y are FITC and BHQ-1, respectively. Binding arms of DNAzymes are underlined. (b) Structural formulas LNA (left) and 2′-OMe (right).

Poly(L-lysine hydrobromide) (PLL-HBr, *M*_w_ = 7.5 × 10^3^) and dextran (Dex, *M*_w_ = 8.0 × 10^3^–1.2 × 10^4^) were obtained from Sigma-Aldrich (St. Louis, MO, USA) and Funakoshi Co. (Tokyo, Japan), respectively. Sodium hydroxide, sodium chloride and manganese(II) chloride tetrahydrate were purchased from Wako Pure Chemical Industries (Osaka, Japan). 2-[4-(2-hydroxyethyl)piperazin-1-yl]ethanesulfonic acid (HEPES) was obtained from Nacalai Tesque, Inc. (Kyoto, Japan). Poly(L-lysine)-*graft*-Dextran (PLL-*g*-Dex) cationic comb-type copolymer was synthesized by a reductive amination reaction of dextran with PLL according to [14]. The resulting copolymer was purified by an ion exchange column and dialysis, and obtained by freeze drying. ^1^H nuclear magnetic resonance spectrometer and gel permeation chromatography equipped with a multi angle light scattering detector were employed to characterize the resulting copolymer. PLL-*g*-Dex copolymer consisting of 10 wt% PLL and 90 wt% dextran (11.5 mol.% of lysine units of PLL were substituted with dextran) was used in this study (Figure 1). HPLC-grade oligonucleotide with LNA modification was purchased from Gene Design Inc. (Osaka, Japan). HPLC-grade oligonucleotides with the sequences summarized in Figure 2(a) except that with LNA modification were purchased from Fasmac Co., Ltd (Kanagawa, Japan) and used without further purification.

### 2.2. Methods

#### 2.2.1. Förster resonance energy transfer analysis to trace the DNAzyme cleavage reaction

A substrate labeled with fluorescein isothiocyanate (FITC) and BHQ-1 quencher (final concentration: 200 nM) in 50 mM HEPES (pH 7.3), 150 mM NaCl, and 5.0 mM Mn^2+^ was pre-incubated with PLL-*g*-Dex at the ratio of [positively charged amino groups]_copolymer_/[negatively charged phosphate groups]_DNA_ (N/P ratio) of 2 at the reaction temperature for 5 min in a quartz cell. DNAzyme reaction was initiated by injecting DNAzyme solution (final concentration: 0.25 or 2.0 nM) into the cell. The fluorescence intensity of the solution was acquired using a fluorescence spectrometer (FP-6500 Jasco, Tokyo, Japan) at an excitation wavelength, λ_ex_, of 494 nm and an emission wavelength, λ_em_, of 520 nm, respectively, with excitation and emission slits at 3 nm. The fluorescence intensity curve over time was used to fit the following equation.

$$I_{t}=I_{0}+\;\left(I_{\infty }-I_{0}\right)\;\left(1\;-e^{-k}{\text{obs}}^{t}\right)$$

where *I*_*t*_ was the fluorescence intensity at any reaction time *t*, *I*_∞_ was the fluorescence intensity after incubating at 50°C for 24 h, *I*_0_ was the initial fluorescence intensity (background).

Initial period of the cleavage reaction curve was fitted to estimate the *k*_obs_ values.

#### 2.2.2. Melting temperature measurement

DNAzyme and substrate (1.0 μM in final concentration) were mixed in a 50 mM HEPES containing 150 mM NaCl (pH 7.3) in the absence or presence of PLL-*g*-Dex (N/P = 2). The mixture was heated at 95°C for 5 min, followed by subsequent slow cooling to room temperature over 12 h. UV-*T*_m_ curves were obtained at 260 nm on V-630 spectrophotometer (Jasco) at heating rate of 1.0°C min^–1^ from 25 to 95°C. Melting data were collected and fitted with Spectra Manager (Jasco).

## 3. Results and discussion

**Figure 3.**
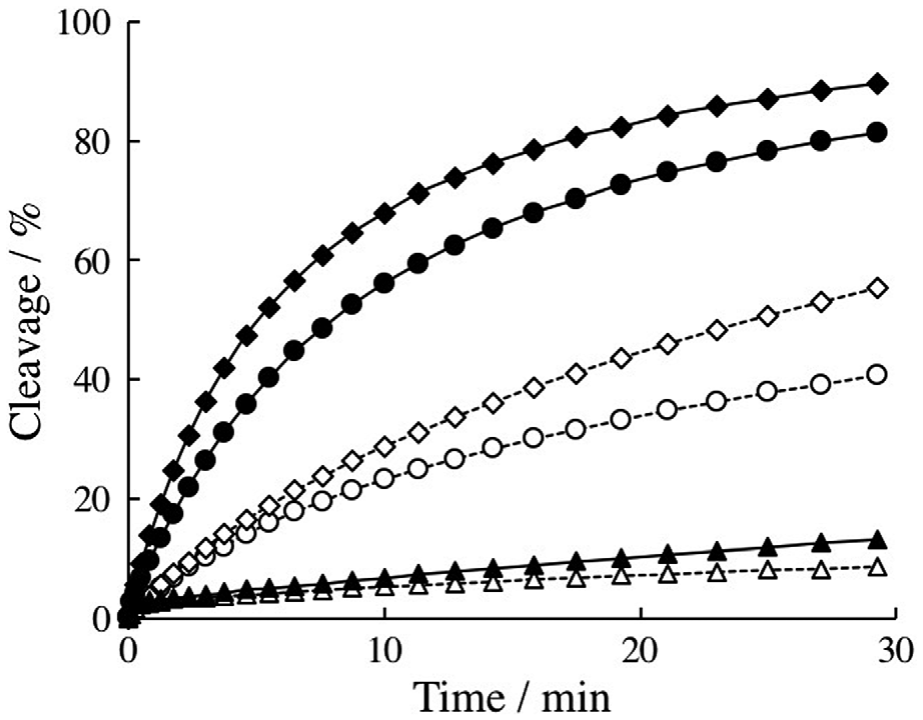
Cleavage over time profiles of unmodified (circle), 2′-OMe (square) and LNA modified (triangle) 10–23 DNAzymes under multi-turnover reaction conditions in the absence (dotted line) or presence PLL-*g*-Dex (N/P = 2) (solid line) at 35°C. Initial substrate conc.:100 nM, DNAzyme concentration: 2.0 nM.

We observed DNAzyme reaction in the absence or presence PLL-*g*-Dex at 35°C for 30 min under multiple-turnover condition, [S] / [E] = 50. The results are shown in Figure 3. While LNA modification significantly decreased DNAzyme reaction rate, 2′-OMe modification increased it.

As reported previously, the cationic comb-type copolymer increased the reaction rate of unmodified DNAzyme. Similar accelerating effect of the copolymer was observed for 2′-OMe modified DNAzyme. The accelerating effect of the copolymer on LNA-modified DNAzyme was also observed but its effect was not considerable compared to those observed with unmodified or the LNA-modified one.

**Figure 4.**
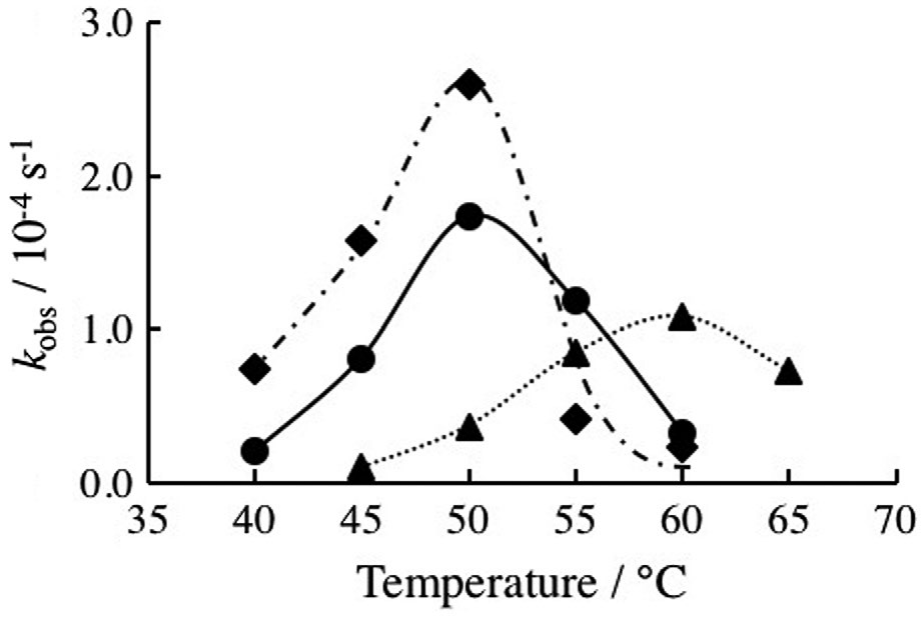
Temperature dependence of rate constants, *k*_obs_, estimated in the absence of the copolymer: unmodified (circle), 2′-OMe- (square) and LNA-modified 10–23DNAzyme (triangle).

**Figure 5.**
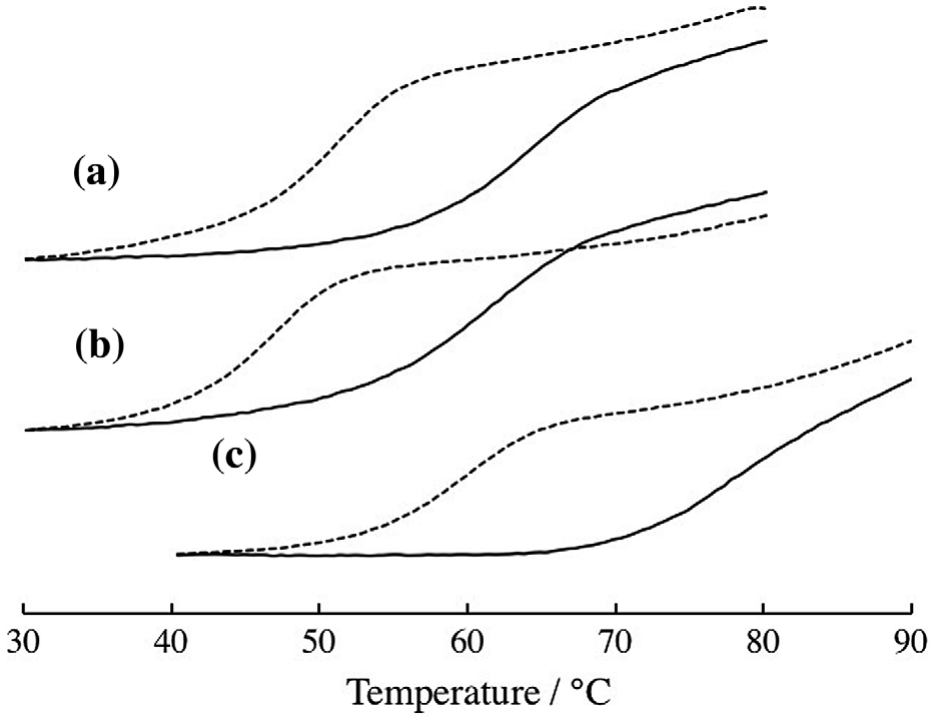
UV-melting curves of DNAzyme/substrate complexes in the absence (dotted line) and presence (solid line) of PLL-*g*-Dex (N/P = 2). (a) Unmodified; (b) 2′O-Me-modified; and (c) LNA-modified 10–23 DNAzymes.

**Scheme 1.**
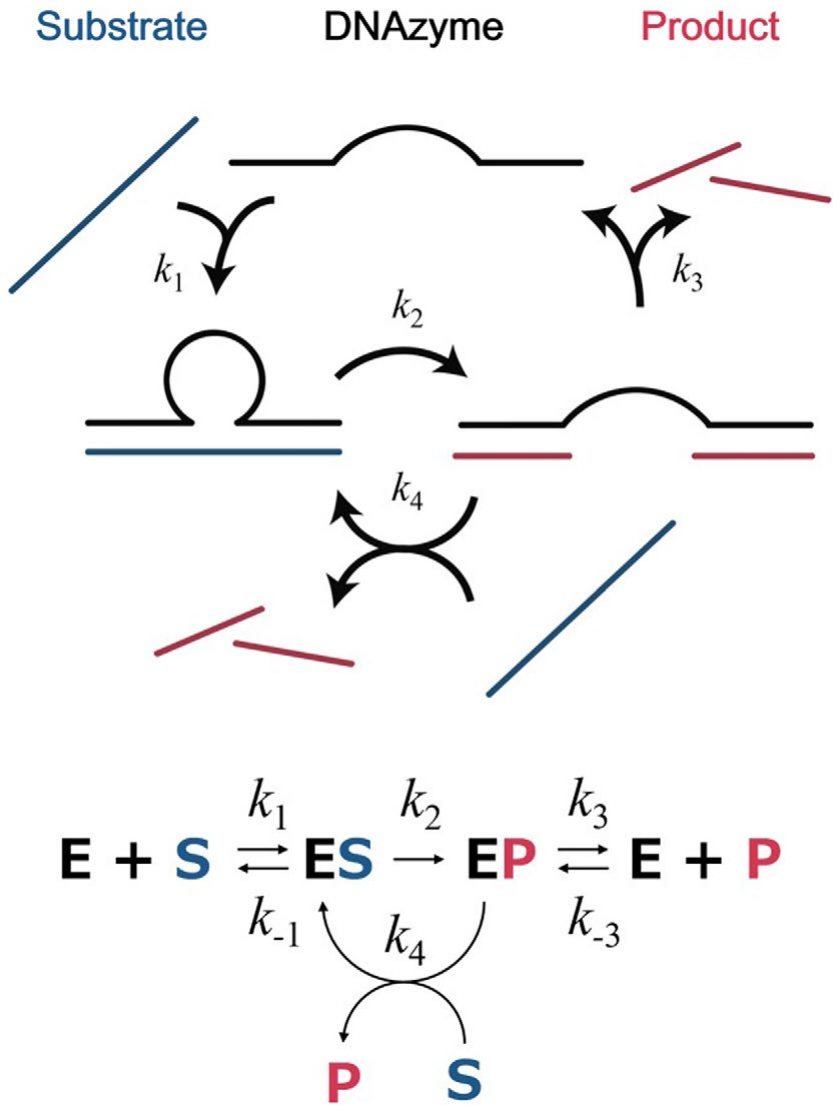
Schematic representation of DNAzyme reaction pathways.

Chemical modification of DNAzyme at its substrate binding arms influences stability of enzyme/product complex and enzyme/substrate complex, so that the optimum temperatures for DNAzyme reactions should be influenced by the modification. To estimate influence of the chemical modification on the optimum temperatures, temperature dependences of DNAzyme activities were estimated. As shown in Figure 4, the unmodified DNAzyme has an optimum temperature at 50°C. While the 2′-OMe modification did not significantly alter the optimum temperature it increased the DNAzyme activity. The LNA-modified DNAzyme has an optimum temperature at 60°C, indicating significant increase in the optimum temperature. The LNA-modified DNAzyme activity was reduced. We then estimated stability of enzyme-substrate (ES) complexes by measuring their melting temperatures, *T*_m_s. UV-melting curves of ES complex are shown in Figure 5. The values of *T*_m_ determined from Figure 5 and the reaction rates of DNAzymes at the optimum temperatures are summarized in Table 1. Close relationships between the optimum temperature and the *T*_m_ values were shown. The results indicated that DNAzyme activity was not determined by the chemical cleavage step (*k*_2_ in Scheme 1) but hybridization dynamics (*k*_1_ and *k*_3_ and their inverse reaction rates in Scheme 1) of DNAzymes with either its substrates or products (P). In general, with increasing temperature, dissociation rates of DNA hybrids increase while their association rates decrease. The increase in enzymatic activity with increasing temperature up to the optimum temperatures is attributed to the increase in dissociation rate of EP complex. Decrease in the enzymatic activity at temperatures higher than the optimum temperature resulted from a decrease in association rate of the ES complex. We observed a 10°C increase in *T*_m_ for the ES complex with LNA modification. It is well established that that LNA modification increased stability of DNA hybrids.[20,21] In our experimental conditions, LNA modification resulted in approximately 40% loss in the DNAzyme activity. LNA modification stabilized enzyme/product (EP) complex as well as ES complex and reduced dissociation rate (*k*_3_ in Scheme 1) of the EP complex. The decrease in the dissociation rate of the EP complex resulted in slow turnover, leading to the decrease in DNAzyme reactivity under multiple turnover conditions. This consideration was supported by the fact that the LNA modification stabilizes the DNA hybrid by reducing the dissociation rate rather than increasing the association rate of hybridization.[27] LNA modification was considered to positively affect the DNAzyme reactivity by stabilizing the ES complex (i.e. decreasing Michaelis constant *K*_M_) if the reaction is carried out under single-turnover reaction condition. While 2′-OMe modification resulted in a slight decrease in *T*_m_, it increased 1.5 times the catalytic activity compared to that of unmodified DNAzyme when compared at their optimum temperatures. We speculate that 2′-OMe modification decreased *T*_m_ by increasing the dissociation rate of the DNA duplex. The 2′-OMe modification seemingly promoted dissociation of EP complex, resulted in faster multiple-turnover reaction.

**Table 1.** Melting temperatures of ES complexes and enzymatic reaction rates at the optimum temperatures.

DNAzyme	PLL-*g*-Dex N/P ratio	*T*_m_^*^	Δ*T*_m_	*k*_obs_ / s^−1^ at optimum temperature	Relative rate
Unmodified	0	50.0	–	1.73 × 10^−4^ / 50°C	1.0
	2	61.5	11.5	3.03 × 10^−3^ / 60°C	17.5
2′-OMe	0	46.0	−4.0	2.59 × 10^−4^ / 50°C	1.50
	2	58.5	8.5	6.02 × 10^−3^ / 60°C	34.8
LNA	0	59.2	8.2	1.08 × 10^−4^ / 60°C	0.62
	2	71.8	21.8	4.73 × 10^−4^ / 65°C	2.73

^*^ Melting temperature, at which half of the DNAzyme/substrate complex was denatured, was determined from the UV-melting curve in Figure 5 at [DNAzyme] = [substrate] = 1.0 μM in 50 mM HEPES, 150 mM NaCl.

**Figure 6.**
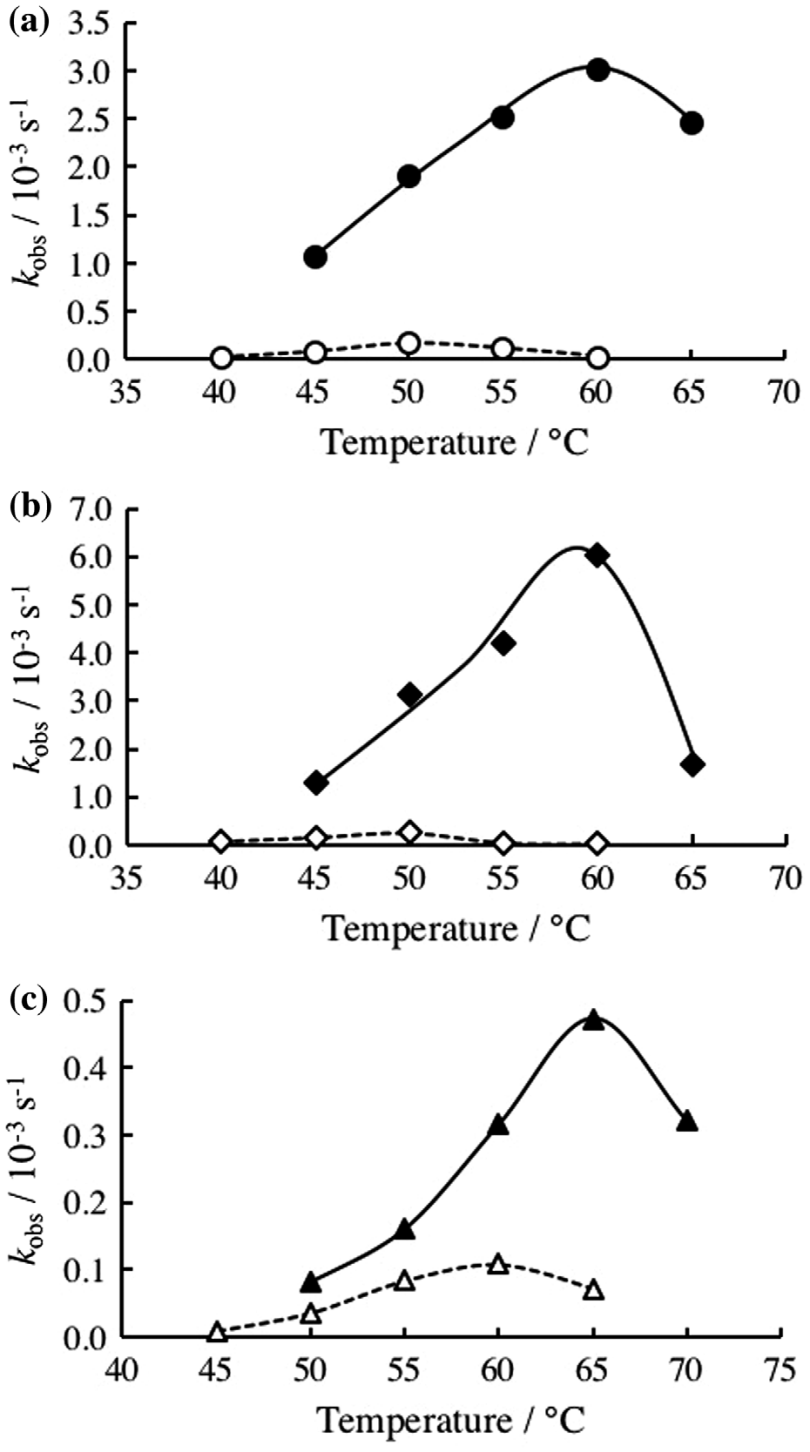
Temperature dependence of rate constants, *k*_obs_, estimated in the absence (dotted line) and presence (solid line) of the copolymer. (a) Unmodified; (b) 2′-OMe-modified; and (c) LNA-modified 10–23 DNAzymes.

We then assessed the influence of added PLL-*g*-Dex copolymers on unmodified and chemically modified DNAzymes (Figure 6 and Table 1). The copolymer increased more than 17-fold the catalytic activity of DNAzymes at the optimum temperature, in accordance with our previous observation.[18] The copolymer increased *T*_m_ of the ES complex by 11.5°C. The added PLL-*g*-Dex increased *T*_m_ of the ES complex similarly to LNA-modification, indicating both the added PLL-*g*-Dex and the LNA modification contributed to the stabilization of ES complex. Stability of EP complex should also be increased by the added PLL-*g*-Dex as well as the LNA modification. In contrast to the LNA modification that stabilizes the DNA hybrid by decreasing a dissociation rate of a DNA hybrid, the copolymer does it principally by increasing its association. The copolymer likely increases the turnover step by promoting ES complex formation whereas the LNA modification retards it by decreasing the dissociation rate of the EP complex.

It is noted that the highest catalytic activity was observed with 2′-OMe modification in the presence of the copolymer. More than 30-fold enhancement in multiple-turnover reactivity was found. The added copolymer and 2′-OMe modification contributed the diverse steps, i.e. *k*_1_ and *k*_3_, respectively, in the turnover process, so that their activities are well harmonized. In fact, the 30-fold enhancement achieved by the copolymer and 2′-OMe modification are nearly consistent with the product of 1.5-fold and 17.5-fold enhancements that were observed for 2′-Me modification and the added copolymer, respectively. The copolymer was also effective to enhance activity of LNA-modified DNAzyme, although the magnitude of the enhancement is moderate. LNA-modified DNAzyme showed considerably stronger activity than the unmodified one when substrates had intramolecular structures.[28] Strong hybridization ability of LNA-modified binding arms permitted invasion of the DNAzyme to the structured substrates. Our result suggested that the copolymer in combination with LNA modification would be useful to enhance DNAzyme activity toward structured targets. Our previous study indicated that the copolymer further enhanced hybridization property of LNA modified oligonucleotide.[29] The copolymer promoted DNA/LNA assembly as well as DNA/DNA assembly by reducing the counterion condensation effect as estimated by thermodynamic analysis.[29]

As seen from Figure 5, the copolymer considerably increased enzymatic activity of DNAzymes examined over a wide temperature range. Below the optimum temperature, the rate-determining step is the dissociation of the EP complex, so that the copolymer was thought not to be effective to accelerate the reaction by promoting ES complex formation. Nevertheless, obvious accelerating effects of the copolymer were observed even at a temperature significantly lower than the optimum temperature. It would be possible for the copolymer to facilitate a turnover process by the strand exchange pathway (*k*_4_ of Scheme 1) of EP complexes with substrates to form fresh ES complex, because the copolymer is capable of activating strand exchange reactions between double stranded DNA and its homologous single strand DNA.[30,31]

## 4. Conclusions

In this study, we showed that DNAzyme catalytic activity was successfully enhanced by cooperation of the added copolymer and chemical modification of DNAzyme. The cationic comb-type copolymer, PLL-*g*-Dex, increased multiple-turnover reactivity of chemically modified DNAzymes as well as an unmodified DNAzyme. Thirty-fold enhancement of the activity was achieved with 2′-OMe modification in the presence of the copolymer. Kinetic effects rather than thermodynamic effect of the chemical modifications and the copolymer on DNA hybridization likely play a pivotal role in the observed cooperative effect.

## Disclosure statement

No potential conflict of interest was reported by the authors.

## Funding

Parts of this work were supported by a Grant-in-Aid for Scientific Research on Innovative Areas ‘Molecular Robotics’ [number 15H00804], ‘Nanomedicine Molecular Science’ [number 2306] and the Cooperative Research Program of ‘Network Joint Research Center for Materials and Devices’ from the Ministry of Education, Culture, Sports, Science and Technology, by Center of Innovation (COI) Program, Japan Science and Technology Agency (JST), and by KAKENHI [number 15H01807, 25350552] from Japan Society for the Promotion of Science.
